# Dandy-Walker malformation associated with massive occipital encephalocele: A case report

**DOI:** 10.1016/j.ijscr.2025.111612

**Published:** 2025-07-07

**Authors:** Allahdad Khan, Nehan Zahoor, Noor ul Ain Saleem, Humaira Siddique, Asma Sattar, Jamil Nasrallah

**Affiliations:** a Department of Medicine, Nishtar Medical University, Multan, Pakistan; b Department of Neurosurgery, Nishtar Medical University, Multan, Pakistan; c Department of Medicine, FMH College of Medicine and Dentistry, Lahore, Pakistan; d Department of Radiology, Nishtar Medical University, Multan, Pakistan; e Department of Medicine, Faculty of Medical Sciences, Lebanese University, Beirut, Lebanon

**Keywords:** Dandy-Walker malformation, Occipital encephalocele, Occipital meningocele, Case report

## Abstract

**Introduction:**

Dandy-Walker malformation (DWM) is a rare congenital anomaly of the posterior fossa characterized by vermian hypoplasia, cystic dilation of the fourth ventricle, and hydrocephalus. Occipital encephalocele, a neural tube defect involving herniation of meninges and brain tissue, is an uncommon association with DWM, occurring in less than 5 % of cases. The coexistence of these anomalies presents significant diagnostic and management challenges.

**Case presentation:**

We report the case of a one-month-old female infant of Pakistani origin who presented with a large occipital cystic swelling since birth and poor feeding. Clinical examination revealed macrocephaly, a soft and transilluminating occipital cyst, decreased muscle tone, and diminished reflexes. Neuroimaging confirmed features of DWM, including vermian hypoplasia, a posterior fossa cyst communicating with the fourth ventricle, and hydrocephalus, along with a large occipital encephalocele communicating with the cyst. The patient underwent surgical excision of the encephalocele, followed by ventriculoperitoneal shunting for hydrocephalus. Postoperative recovery was stable, with resolution of hydrocephalus following shunt placement.

**Discussion:**

The patient was discharged with recommendations for regular follow-up to monitor neurological development and potential complications. DWM associated with occipital encephalocele is an extremely rare condition that requires early diagnosis and multidisciplinary management.

**Conclusion:**

Surgical intervention, including encephalocele excision and cerebrospinal fluid diversion, plays a crucial role in improving outcomes. Long-term follow-up is essential to assess neurological development and manage potential complications. This case highlights the importance of timely intervention and individualized treatment strategies for such complex congenital anomalies.

## Highlights

• This case presents a rare coexistence of Dandy-Walker malformation (DWM) with occipital encephalocele, an anomaly observed in less than 5 % of DWM cases.
• Neuroimaging confirmed characteristic features of DWM, including vermian hypoplasia, a posterior fossa cyst communicating with the fourth ventricle, and an associated occipital encephalocele.
• The patient underwent successful surgical excision of the encephalocele, followed by ventriculoperitoneal shunting to manage postoperative hydrocephalus.
• The patient demonstrated clinical stability following intervention, with hydrocephalus successfully resolved after shunt placement.
• This case highlights the need for a multidisciplinary approach, early neuroimaging, and timely surgical intervention for optimal management of complex congenital CNS anomalies.

## 1 Introduction

Dandy-Walker malformation and related variants occur in approximately 1 in 30,000 live births [1]. It is more common in females. Central nervous system malformations outside of the syndrome are observed in about 70 % of the patients, out of which occipital encephalocele is detected in less than 5 % of cases [2]. Encephaloceles represent a distinct central nervous system defect that causes brain tissue surrounded by meninges or cerebrospinal fluid to push through a hole in the skull bones with varying neurological outcomes [3]. The joint occurrence of these two anomalies is not frequent in medical literature. In this case report we present a rare case of dandy walker malformation associated with occipital encephalocele in a one-month-old infant. This case report is reported in the line of SCARE guidelines [4].

The patient gave consent implicitly to share this case report with images through publication. The patient received assurances about full confidentiality of identifying information while researchers stated that their findings will only serve academic and educational needs and future patient care improvement through similar cases.

## 2 Case presentation

A one-month-old female patient of Pakistani origin presented to the outpatient department with complaints of large cystic swelling in the occipital region of the head since birth and poor feeding one week after birth. The patient was born uneventfully through cesarean section delivery at 38 weeks of gestation. During pregnancy, the patient's mother took adequate prenatal care with folic acid supplements prescribed by her gynecologist. There was no history of congenital anomalies in the family.

**Fig. 1:**
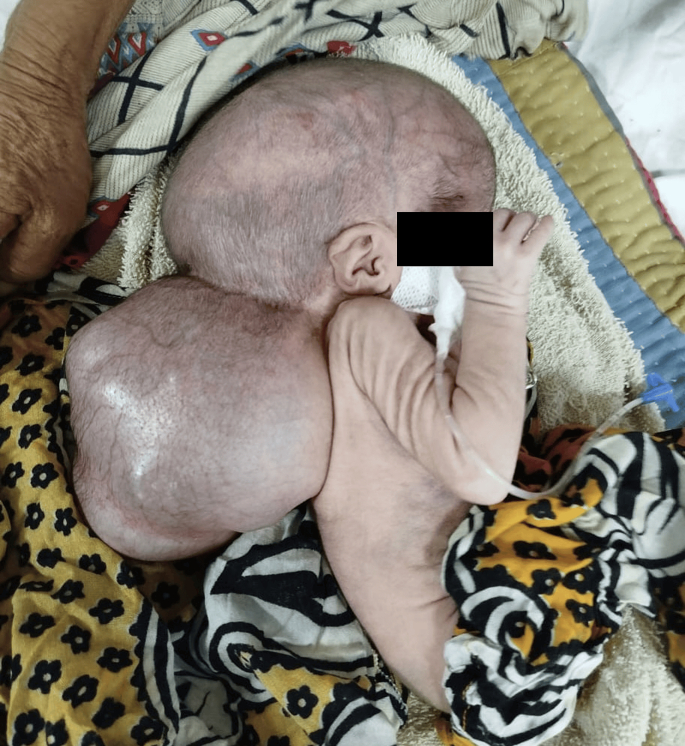
Presentation of the large cyst in the occipital region of the patient.

On examination, there was macrocephaly with a giant cyst in the occipital region measuring approximately 15 × 12 cm (Fig. 1) that was soft to the touch and transilluminated. The overlying skin was thin but intact, with no discoloration or ulceration, and showed visible dilated veins. There was no bruit or thrill noted over the swelling. The mass was non-pulsatile and non-expansile, and no signs of infection or inflammation were present. The patient had decreased muscle tone with decreased reflexes. Her head was not under control.

**Fig. 2:**
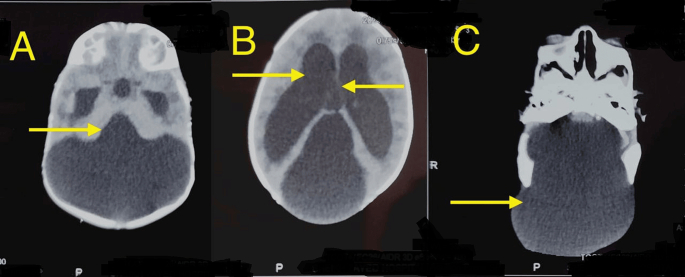
CT brain plain axial sections (A) shows large cystic area occupies the posterior fossa communicating directly with the fourth ventricle associated with vermian hypoplasia (representing Dandy walker malformation) and moderate dilatation of the lateral and third ventricles (B). This large cyst shows communication with an occipital encephalocele protruding through bone defect (C).

All laboratory investigations were normal. CT brain plain axial sections (Fig. 2) showed a large cystic area that occupies the posterior fossa communicating directly with the fourth ventricle associated with vermian hypoplasia (representing Dandy walker malformation) and moderate dilatation of the lateral and third ventricles. The large cyst showed communication with an occipital encephalocele protruding through a bone defect.

**Fig. 3:**
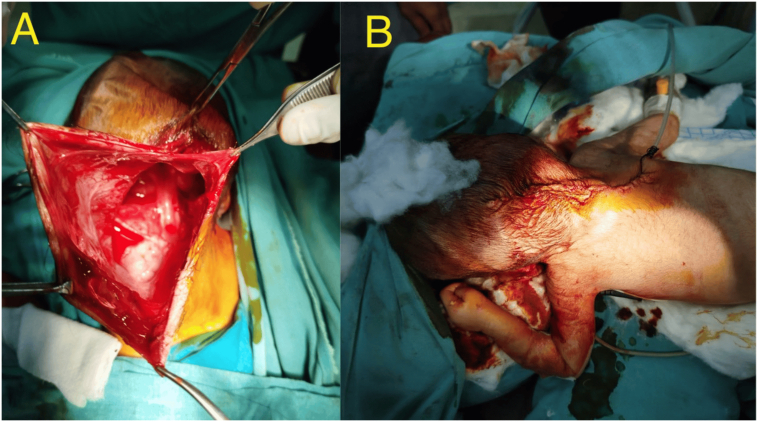
A: Intraoperative surgical excision of encephalocele. B: Postoperative stitching of the patient after excision of encephalocele.

Surgical excision of encephalocele (Fig. 3) was done under general anesthesia. Intraoperatively, the encephalocele sac measured approximately 15 × 12 cm and was noted to have a thin, translucent wall. The sac contained clear, straw-colored cerebrospinal fluid (CSF) without any signs of hemorrhage, cloudiness, or infection. There were no internal septations noted, and no neural elements were identified within the sac.

Following surgery, the patient was transferred to the Neonatal Intensive Care Unit (NICU) for close monitoring. She remained hemodynamically stable, and there were no immediate postoperative complications. The patient was discharged on postoperative day 7, with sutures removed on day 10 in the outpatient setting.

At the 10-day postoperative follow-up, the patient had developed signs of progressive hydrocephalus, including increasing head circumference, bulging fontanelle, and irritability. A ventriculoperitoneal shunt was placed to divert CSF and relieve intracranial pressure. The procedure was successful, and the patient's symptoms improved postoperatively. She was then recommended for regular visits to track her progress.

## 3 Discussion

Dandy-Walker malformation (DWM) is a rare congenital posterior fossa anomaly that is characterized by an expanded posterior fossa, cystic dilatation of the fourth ventricle, and hypoplasia or agenesis of the cerebellar vermis. The frequency is estimated to be 1 in 30,000 live births, making it the most prevalent posterior fossa malformation [1]. The neural tube defect known as occipital encephalocele, on the other hand, occurs when meninges and brain tissue herniate through an occipital bone defect. Occipital encephalocele with DWM co-occurring is extremely uncommon**; only a few examples have been** documented in the literature [5]. Since these two anomalies occur simultaneously, this case is significant because it illustrates the difficulties in diagnosing and treating them. Although hydrocephalus and other disorders of the central nervous system are frequently linked to DWM, its relationship to occipital encephalocele is less clear and does not have established therapy guidelines [5]. The estimated prevalence of Dandy-Walker malformation (DWM), an uncommon congenital defect, is between 1 in 25,000 and 35,000 live births. It makes up around 7.5 % of cases of infantile hydrocephalus and is the most prevalent posterior fossa abnormality [6]. The female-to-male ratio can reach 3:14, meaning that the disease is more common among females. Agenesis of the corpus callosum, occipital encephalocele, and hydrocephalus, which affects 80–90 % of cases, are among the additional central nervous system (CNS) abnormalities that are frequently linked to DWM [7]. Although some people may have normal cognitive and motor development, others may have severe neurodevelopmental delays, motor impairments, and behavioral challenges. Hydrocephalus is a common complication of DWM that frequently necessitates surgical intervention such as ventriculoperitoneal shunting. The presence of additional CNS or systemic anomalies influences long-term outcomes [8].

Our patient's MRI results showed vermian hypoplasia, significant dilatation of the lateral and third ventricles, and a posterior fossa cyst communicating with the fourth ventricle—all of which are hallmarks of DWM. The intricacy of the case was further highlighted by the occipital encephalocele, which was a distinguishing characteristic with an uncommon communication between the cyst and the encephalocele. However, our kid's presentation at one month of age underlines early clinical detection, maybe because of the apparent occipital enlargement, whereas Sule's instance appeared in a male patient who was 10 months old [9].

The lateral and third ventricles in our case had moderate dilatation with vermian hypoplasia, whereas another case report shows cystic dilatation of both ventricles with herniation due to a bone abnormality, which was validated prenatally by ultrasonography. Long-term results are another difference; our patient's hydrocephalus was successfully managed after surgery, while the new case revealed issues like nystagmus, tremors, and epilepsy during follow-up. Both cases involve sac excision and ventriculoperitoneal shunting for hydrocephalus, which is comparable in surgical therapy despite these variances [10]. Bindal et al. reviewed a personal series of 50 DWM cases in 1991 and found eight cases associated with OMC. Although they only found 11 further occurrences in the worldwide literature, these authors predicted that 16 % of DWM would have this association [11]. Agarwal and Thakur reported a case with posterior fossa abnormalities and macrocephaly, along with imaging findings such as vermian hypoplasia and a posterior fossa cyst. A distinguishing feature of our case, absent in the previously reported instance, was the presence of an occipital encephalocele with cystic communication. Additionally, compared to the delayed development observed in the other case, our patient's quick advancement to hydrocephalus within 10 days after surgery indicates a more aggressive clinical course. These variations highlight the range of presentations and results, underscoring the significance of tailored diagnostic and treatment strategies [12].

Both occipital encephalocele and DWM have intricate and connected embryological pathways. According to Spennato et al., DWM is believed to be caused by defective hindbrain development between weeks 7 and 10 of pregnancy, which results in vermian hypoplasia and the creation of posterior fossa cysts [13]. On the other hand, occipital encephalocele, a neural tube deformity, is thought to result from incorrect closure of the cranial neuropore, which may be impacted by genetic predispositions and folate metabolism. Given that both disorders entail early developmental disturbances, it is conceivable that genetic factors contribute to their co-occurrence [14].

Numerous mutations linked to posterior fossa abnormalities have been found through genetic research, including those involving the cerebellar development regulators FOXC1 and ZIC1 [15]. Even though no particular genetic testing was done in this instance, taking into account genetic screening for comparable presentations could help determine familial risk and the chance of recurrence.

The key to treating hydrocephalus, a frequent consequence, is surgical intervention, such as ventriculoperitoneal (VP) shunting. By redirecting cerebrospinal fluid (CSF), shunting successfully lowers intracranial pressure and stops increasing neurological impairment [16]. To safeguard the brain and spinal structures and lower the danger of infection and additional disability, surgical excision and repair are carried out in cases when concomitant anomalies, such as occipital encephalocele, are present [17]. The significance of long-term follow-up care is highlighted by postoperative monitoring, which guarantees early detection and treatment of problems such as shunt malfunction. Physical, occupational, and speech therapy are examples of supportive therapies that help improve motor, cognitive, and communication skills as well as address developmental delays [18,19]. Furthermore, complete management that addresses both short-term demands and long-term development is ensured by multidisciplinary care coordinated by neurosurgeons, neurologists, and therapists. Better health results and a higher quality of life are made possible by this comprehensive strategy, which dramatically improves the patient's condition.

The case report provides a detailed clinical account of a one-month-old patient with Dandy-Walker malformation and occipital encephalocele. However, several weaknesses limit its academic depth and clarity. The lack of genetic testing, the very little follow-up time, and the absence of prenatal imaging, which would have offered early diagnostic insights are missing.

Another limitation is the minimal discussion on family and genetic history. While there is no known family history of congenital anomalies, the report does not consider whether genetic counseling or testing was recommended, which is particularly relevant for cases involving structural brain malformations. A more comprehensive assessment of potential perioperative challenges and postoperative recovery would strengthen the clinical relevance of the report. Future studies should evaluate the best surgical techniques for patients with dual abnormalities and investigate long-term neurodevelopmental results. A multidisciplinary approach involving neurosurgeons, geneticists, and developmental specialists is essential for comprehensive management (Table 1).

**Table 1:** Key differences.

Feature	Previously reported cases	Present case
**Age at presentation**	Typically between 5 and 10 months of age	1 month, indicating earlier clinical detection due to visible occipital enlargement
**Sex distribution**	Male predominance in some reports (e.g., Sule et al.)	Female, in keeping with DWM's documented female predominance
**Imaging findings**	Posterior fossa cyst, vermian hypoplasia, hydrocephalus; occipital encephalocele reported but not always communicating with the cyst	An uncommon and underreported characteristic is the direct anatomical contact between the occipital encephalocele and the posterior fossa cyst.
**Sac contents**	Some cases report neural elements or hemorrhagic content (e.g., Mishra et al.)	The absence of septations, hemorrhage, neural tissue, and clear CSF all point to a better surgical profile.
**Timing and sequence of surgery**	Often delayed or staged; some cases lack detailed perioperative timelines	Proactive and organized management is demonstrated by the prompt VP shunt implantation within 10 days of the surgical resection of encephalocele.
**Postoperative course**	Variable; some report complications like seizures, nystagmus, or developmental delays	Early clearance of hydrocephalus following shunting, no acute problems, and rapid follow-up
**Prenatal and perinatal history**	Often incomplete or lack folate supplementation data	Prenatal care with a solid record, folic acid supplements, and no family history—all contribute to the value of etiological considerations.
**Genetic evaluation**	Rarely performed or reported	The case recognizes the necessity of genetic screening, even though it is not carried out here, showing awareness of evolving standards
**Clinical implication**	Most reports emphasize rarity but lack structured treatment pathways	Our case provides a possible paradigm by illustrating a distinct diagnostic-to-treatment continuum **for early intervention** in similar presentations.

## 4 Conclusion

This case report illustrates the rare co-occurrence of Dandy-Walker malformation and occipital encephalocele in a one-month-old infant, emphasizing the challenges in diagnosis and treatment. Successful surgical intervention, including encephalocele excision and ventriculoperitoneal shunting for hydrocephalus, highlights the potential for positive outcomes with timely care. Despite the limitations of not having genetic testing and prenatal imaging, the case contributes to the literature on this uncommon combination of abnormalities. Further research into genetic factors, optimal surgical approaches, and long-term outcomes is needed to improve patient care and prognosis.

## Patient or volunteer consent

Written informed consent was obtained from the patient for publication of this case report and accompanying images.

## CRediT authorship contribution statement

A.K., N.Z., N.S., and H.S.: conceptualized the study, curated data, and conducted the investigation; A.K., N.S., A.S. and H.S.: wrote the original manuscript; A.K., N.Z.: reviewed and edited the manuscript; A.K., and J.N.: administered the project; J.N.: supervised the study.

## Consent

Written informed consent was obtained from the patient's parents/legal guardian for publication and any accompanying images. A copy of the written consent is available for review by the Editor-in-Chief of this journal on request.

Research Registration (for case reports detailing a new surgical technique or new equipment/technology): Not applicable.

## Ethical approval

Ethical approval is not required for the case report in our institution.

## Guarantor

Jamil Nasrallah.

## Sources of funding

No funding was received.

## Declaration of competing interest

The authors declare no potential conflicts of interest with respect to the research, authorship, and/or publication of this article.

## Data Availability

All the relevant data have been included in the manuscript itself.
